# Photosystem II photoinhibition and photoprotection in a lycophyte, *Selaginella martensii*

**DOI:** 10.1111/ppl.13604

**Published:** 2021-12-06

**Authors:** Andrea Colpo, Costanza Baldisserotto, Simonetta Pancaldi, Alessandra Sabia, Lorenzo Ferroni

**Affiliations:** ^1^ Department of Environmental and Prevention Sciences University of Ferrara Ferrara

## Abstract

The Lycophyte *Selaginella martensii* efficiently acclimates to diverse light environments, from deep shade to full sunlight. The plant does not modulate the abundance of the Light Harvesting Complex II, mostly found as a free trimer, and does not alter the maximum capacity of thermal dissipation (NPQ). Nevertheless, the photoprotection is expected to be modulatable upon long‐term light acclimation to preserve the photosystems (PSII, PSI). The effects of long‐term light acclimation on PSII photoprotection were investigated using the chlorophyll fluorometric method known as “photochemical quenching measured in the dark” (qP_d_). Singularly high‐qP_d_ values at relatively low irradiance suggest a heterogeneous antenna system (PSII antenna uncoupling). The extent of antenna uncoupling largely depends on the light regime, reaching the highest value in sun‐acclimated plants. In parallel, the photoprotective NPQ (pNPQ) increased from deep‐shade to high‐light grown plants. It is proposed that the differences in the long‐term modulation in the photoprotective capacity are proportional to the amount of uncoupled LHCII. In deep‐shade plants, the inconsistency between invariable maximum NPQ and lower pNPQ is attributed to the thermal dissipation occurring in the PSII core.

## 1 INTRODUCTION

The evolution of the photosynthetic apparatus allowed land plants to adapt to a broad range of light conditions, from extreme shade to full sunlight. However, any change in light regime during the plants' lifetime represents a major threat to their survival and requires structural and functional adjustments of their photosynthetic machinery (developmental acclimation) (Lichtenthaler et al., 2007; Pribil et al., 2014; Ruban et al., 2012).

*Selaginella martensii* Spring is a shade plant typical of the understory of tropical and equatorial rainforests. However, this ancient tracheophyte is sufficiently flexible to acclimate to extreme light regimes, such as deep shade or full sunlight (Ferroni et al., 2016; Ferroni, Brestič, et al., 2021). Its long‐term acclimation to different light regimes produces major rearrangements in the thylakoid organization and photosystem I (PSI) and II (PSII) relative abundance, whereas, unlike most angiosperms, it does not modulate the light‐harvesting antenna complex II (LHCII) content and the total thermal dissipation capacity of absorbed excess energy (Ferroni et al., 2016).

Deep‐shade (L) acclimated thylakoids of *S. martensii* are characterized by a peculiar pseudo‐lamellar organization, while both mid‐shade (M) and full‐sunlight (H) plants display a predominant granal structure. The PSI/PSII ratio increases from L to H plants because the PSI content rises in parallel to the increasing light availability, while PSII is more abundant in L and M plants than H. In contrast, the relative amount of LHCII does not change in response to light acclimation (Ferroni et al., 2016). This characteristic seems typical of seedless plants (Gerotto et al., 2011), while angiosperms generally cope with increasing light availability by decreasing the LHCII content (Albanese et al., 2019; Ballottari et al., 2007; Flannery et al., 2021; Schumann et al., 2017). However, despite the invariable LHCII content, the long‐term light acclimation in *S. martensii* strongly influences the LHCII association with PSII. PSII‐LHCII supercomplexes are not high in abundance in native gels of *S. martensii* thylakoids, but the amount is clearly higher in L and M than in H plants (Ferroni et al., 2014, 2016). Higher abundance of PSII‐LHCII supercomplexes in L plants responds to the need for a larger PSII antenna to enhance the harvesting process under limiting light conditions. In contrast, H plants conceivably need a smaller PSII antenna because the light availability is not limiting, and the safe management of excess light is instead the priority. In fact, in *S. martensii* the great majority of LHCII antennae do not form stable complexes with PSII but are found in the form of free trimers (Ferroni et al., 2016). Free LHCII trimers are common in *Viridiplantae*, and their function is a hot topic in photosynthesis research, being possibly involved in thermal dissipation of excess absorbed energy (Holzwarth et al., 2009; Horton et al., 2005; Johnson et al., 2011; Nicol et al., 2019; Shukla et al., 2020), PSII connectivity (Haferkamp et al., 2010; Zivcak et al., 2014), PSI‐PSII interconnectivity (Grieco et al., 2015; Wientjes et al., 2013; Wood & Johnson, 2020). Moreover, the thylakoid membrane of *S. martensii* is characterized by permanent megacomplexes comprised of PSII, PSI, and LHCII, which presence increases from L to H plants (Ferroni et al., 2016). The abundance of these megacomplexes is regulated in response to a short‐term high‐light exposure; in particular, their increase suggests a facilitating role for the energy repartition between PSI and PSII through a mechanism of energy spillover (Ferroni et al., 2016; Yokono et al., 2015).

Non‐photochemical quenching (NPQ) is an operative parameter in fluorescence analysis quantifying the decrease in maximum fluorescence of PSII (F_m_) in the dark‐acclimated state to a lower value F_m_′ in the light‐acclimated state (Bilger & Björkman, 1990). NPQ is due to a series of light‐induced dissipative processes in competition with PSII photochemistry, and, in general, NPQ can be divided into photoprotective and photoinhibitory quenching components. The main photoprotective component is qE, the high energy‐dependent quenching caused by the onset of the transthylakoid ΔpH and upregulated by PsbS activity and zeaxanthin formation (see for review Ruban, 2016). The other minor NPQ components are related to a sustained violaxanthin de‐epoxidation to zeaxanthin (qZ), state transitions linked to phosphorylated LHCII movement from PSII to PSI (qT), light avoidance chloroplast movements (qM) and plastid lipocalin‐dependent antenna quenching qH (see for review Malnoë, 2018; Roach & Krieger‐Liszkay, 2014). The photoinhibitory component is qI, which depends on the thermal dissipation occurring at the photoinactivated PSII (Aro et al., 1993; Demmig‐Adams et al., 2012). In angiosperms, the total NPQ amplitude is mostly due to its qE component and modulated in response to the light environment, increasing from shade to sun plants (Ballottari et al., 2007; Demmig‐Adams, 1998; Demmig‐Adams et al., 2015; Mishra et al., 2012; Schumann et al., 2017; Stewart et al., 2015). Accordingly, angiosperms grown under high light are characterized by a higher photoprotective capacity compared to the shade‐grown (Mathur et al., 2018; Wilson & Ruban, 2020a). Conversely, *S. martensii* plants display a high and invariable total NPQ amplitude, particularly qE amplitude and PsbS content are the same regardless of the light acclimation history of the plant (Ferroni et al., 2016; Ferroni, Brestič, et al., 2021). Nevertheless, there is no evidence whether the PSII photoprotective fraction of NPQ, which prevents PSII photoinactivation, could similarly be independent of long‐term light acclimation in *S. martensii*.

Upon exposure to intense light, PSII photoinactivation can be quantified destructively by monitoring the degradation rate of the D1 PSII core protein (Aro et al., 1993; Kato et al., 2012; Keren et al., 1995) or by the light‐saturated oxygen evolution of PSII in the presence of an artificial electron acceptor (Delieu & Walker, 1983; Mattila et al., 2020; Öquist et al., 1992; Schansker & van Rensen, 1999); however, it is more easily and precisely analyzed in vivo as the decline of PSII photochemical quantum yield (Campbell & Tyystjärvi, 2012; Chow et al., 1991; Mattila et al., 2020; Schansker & van Rensen, 1999) and/or the persistence of a sustained NPQ fraction in darkness (Demmig‐Adams et al., 2012; Nilkens et al., 2010). Ruban and Murchie (2012) proposed an alternative, fast and non‐invasive method to monitor the PSII photoinactivation. Their chlorophyll fluorescence approach is based on the calculation of the parameter qP_d_, “*photochemical quenching measured in the dark*.” qP_d_ assesses the onset of PSII photoinactivation by comparing two values of minimum fluorescence (F_0_′): (a) the actual minimum fluorescence measured after a short far‐red stimulation (F_0_′_act_) and (b) the value of F_0_′ calculated according to Oxborough and Baker (1997), which is an estimate of F_0_′ as a function of NPQ (F_0_′_calc_). qP_d_ varies theoretically between 0 and 1; in the absence of photoinactivation, F_0_′_calc_ matches F_0_′_act_, and correspondingly qP_d_ = 1. The occurrence of photoinactivation affects only F_0_′_act_, whereas F_0_′_calc_ does not account for it; hence, F_0_′_calc_ underestimates F_0_′ (i.e., F_0_′_calc_ < F_0_′_act_), and qP_d_ drops consequently below 1. The theoretical lower limit qP_d_ = 0 could be only reached when all the PSII reaction centers are closed and photoinactivated. qP_d_ values are monitored during experiments in which a plant sample is exposed to subsequent steps with increasing irradiance (light curves). Ruban and Murchie (2012) empirically fixed qP_d_ ≤ 0.98 as the threshold to assess the onset of PSII photoinactivation during a light curve. Accordingly, the effectiveness of photoprotection provided by NPQ to PSII corresponds to the last value of NPQ that allows a qP_d_ value above 0.98. This method was developed and broadly validated in Alexander Ruban's Laboratory in the model angiosperm *Arabidopsis thaliana*, including mutants, chemical treatments, and acclimation to contrasting light regimes (Giovagnetti & Ruban, 2015; Ruban & Belgio, 2014; Tian et al., 2017; Townsend et al., 2018; Ware et al., 2015, 2016; Wilson & Ruban, 2019, 2020b). More recently, the qP_d_ method was also applied to other photosynthetic organisms, such as the spring ephemeral *Bertereoa incana*, *Prunus cerasifera*, *Oryza sativa*, and the algal reef builder *Neogoniolithon* sp. (Gefen‐Treves et al., 2020; Lo Piccolo et al., 2020; Okegawa et al., 2020; Wilson & Ruban, 2020a). However, the *phototropin 2* mutant of *A. thaliana*, which is unable to produce light‐avoidance chloroplast movements, was found to be completely insensitive to photoinhibition according to the qP_d_ method (Wilson & Ruban, 2020b), while it is instead known to be extremely prone to photobleaching (Cazzaniga et al., 2013). Consequently, Bassi and Dall'Osto (2021) consider the qP_d_ method insufficiently validated, that is, not always leading to results consistent with independent methods.

In *S. martensii*, the variability in PSII photoprotection is expected from an ecophysiological point of view, but it seems hardly compatible with the constancy of NPQ and qE amplitudes across L, M, and H plants. The use of the qP_d_ method in the lycophyte *S. martensii* may potentially unveil whether the long‐term acclimation to contrasting light regimes influences the PSII photoprotection capacity. Given the recent introduction of the method and the non‐angiosperm plant species, the qP_d_ method was checked for consistency with a direct assessment of PSII quantum yield loss upon light exposure. The present study shows that the photoprotective capacity of NPQ well matches the growth light regime in *S. martensii*. Moreover, the qP_d_ method indicates the relevance of antenna uncoupling in PSII photoprotection, suggesting a physiological role for the abundant and invariable amount of LHCII in ancient vascular plants.

## 2 MATERIALS AND METHODS

### 2.1 Plant material and growth conditions

*Selaginella martensii* Spring (Selaginellaceae) plants were cultivated in a humid greenhouse of the Botanical Garden of Ferrara, Italy (N 44°50′30″, E 11°37′22″), at 25–30°C and subjected to the natural photoperiod. Deep‐shade plants (L) were grown in conditions of natural shade, with light sheltered by upper‐broadleaved plants. During the daytime, the irradiance maximum was below 10 μmol m^−2^ s^−1^ of photosynthetic photon fluence rate (PPFR). A second group of plants (M) was long‐term acclimated to the mid‐shade light regime (PPFR <80 μmol m^−2^ s^−1^), that is, the typical light environment experienced by *S. martensii* in its natural habitat. Finally, high‐light grown plants (H) were exposed to direct sunlight, which provided typically a maximal PPFR higher than 800 μmol m^−2^ s^−1^. Subsequent biochemical and fluorometric analyses were performed on the terminal branches after at least 3 weeks of acclimation to each light regime.

### 2.2 Thylakoid isolation and blue‐native gel electrophoresis

Branches of *S. martensii* plants were dark acclimated for 1 h. Terminal branches were harvested and grinded in an ice‐cold (−20°C) mortar in the presence of a grinding buffer (Järvi et al., 2011). The whole thylakoid isolation was performed according to Järvi et al., 2011. Extracted thylakoids were promptly frozen and stored in liquid nitrogen until use. For quantification of pigments, thylakoids were solubilized in 90% (v/v) acetone buffered with HEPES‐KOH (pH 7.8) and analyzed using a spectrophotometer Ultrospec 2000 (Pharmacia Biotech). Chlorophyll *a* and *b* content were determined according to Ritchie (2006), while Wellburn's equation (Wellburn, 1994) was used to determine the carotenoid content. For electrophoresis, thylakoid solubilization was performed according to Järvi et al. (2011) using 1.5% β‐dodecylmaltoside. Blue‐native gel electrophoresis (BN‐PAGE) was performed according to Järvi et al. (2011), with modifications as in Giovanardi et al. (2018), maintaining the electrophoretic chamber at 0°C.

### 2.3 Chlorophyll fluorescence measurements

Modulated chlorophyll fluorescence was measured using a Walz Junior PAM (Walz) on independent samples previously dark‐acclimated for 30 min. All the measurements were performed in the morning to avoid the presence of photoinhibition, especially in H plants. Light curves were recorded applying a simplified version of the method described by Ruban and Murchie (2012). Before light exposure, minimum (F_0_) and maximum (F_m_) fluorescence levels in the dark were measured with the saturating pulse (SP, 0.6 s) method, and the variable fluorescence was calculated as F_v_ = F_m_ − F_0_. F_v_/F_m_ > 0.75 was imposed as the minimum acceptable value of PSII quantum yield: plants with lower F_v_/F_m_ were excluded from the analysis. This first measurement was followed by 12 steps of actinic light illumination from 25 to 1500 μmol m^−2^ s^−1^, each lasting 5 min to reach quasi‐steady‐state conditions. Maximum fluorescence level of the quenched, light‐acclimated state (F_m_′) was measured at the end of each actinic light step upon applying an SP. The minimum fluorescence level in the light‐adapted state (F_0_′_act_) was determined as the lowest value when applying a 7‐s‐long far red‐light pulse with the actinic light switched off (Van Kooten & Snel, 1990). Quantum yields of actual PSII photochemistry [Y(II)], non‐regulatory energy loss [Y(NO)], and regulatory thermal dissipation [Y(NPQ)] were calculated according to Hendrickson et al. (2004). The 1‐qP parameter was calculated as an indicator of the excitation pressure inside PSII according to Schreiber et al. (1986). F_m_ quenching to F_m_′ following the onset of light‐induced thermal dissipation was quantified using the Stern‐Volmer NPQ parameter (Bilger & Björkman, 1990). NPQ equals the Y(NPQ)/Y(NO) ratio (Ferroni et al., 2014; Lazár, 2015).

In addition to the light curves, induction curves were also recorded at fixed independent irradiances. After the 30‐min dark‐acclimation, the samples were exposed to 19 min of continuous actinic light illumination (24, 45, 65, 90, 125, 190, 285, 420, 625, and 820 μmol m^−2^ s^−1^), each followed by 38 min and 30 s of dark relaxation. F_m_′ and F_0_′ fluorescence levels were measured every minute during the light induction and at intervals with increasing length during the dark relaxation (30 s, 1 min, 2 min, 5 min, 10 min, and 20 min). The PSII quantum yield loss because of photoinhibition, Y(qI), was calculated as the difference between the PSII quantum yields in the dark‐acclimated sample before the induction curve (F_v_/F_m_) and at the end of the dark‐relaxation period.

### 2.4 qP_d_ parameter and photoprotective NPQ quantification

qP_d_ parameter was calculated at the end of each actinic light illumination step according to Ruban and Murchie (2012). Briefly, qP_d_ compares the actual and calculated values of minimal fluorescence of the light‐adapted state samples as it follows:

$${\mathrm{qP}}_{d}=\frac{F_{m}^{\prime }-F_{0\;\mathrm{act}}^{\prime }}{F_{m}^{\prime }-F_{0\;\text{calc}}^{\prime }}$$

where F_0_′_act_ is the measured minimum fluorescence level, and F_0_′_calc_ is the theoretical minimum fluorescence level according to Oxborough and Baker (1997). However, because at low‐medium irradiances qP_d_ was generally found >1, F_0_′_calc_ values were corrected as described by Ware et al. (2015) accounting for uncoupled and loosely coupled PSII antenna, thus obtaining the new estimate of F_0_′_calc_ in the case of a heterogeneous antenna, F_0_′_het_:

$$F_{0\;\mathrm{het}}^{\prime }={\left(\frac{1}{{\mathrm{nF}}_{0}+F}-\frac{1}{F_{m}}+\frac{\left(\mathrm{NPQ}+1\right)\left(\text{mNPQ}+1\right)}{F_{m}\left[n\left(\text{mNPQ}+1\right)+\left(1-n\right)\left(\mathrm{NPQ}+1\right)\right]}\right)}^{-1}$$

where F_0_ and F_m_ are the entry constants and NPQ is the independent variable. *n* (fraction of coupled antenna), *m* (relative NPQ amplitude of the uncoupled antenna), and *F* (relative fluorescence emission of the uncoupled antenna) are fitting parameters. We further define the fraction of uncoupled antenna *U* as 1‐*n*. The fitting procedure was performed using Origin software v. 2020b or 2021 (OriginLab Corporation, USA). F_0_′_het_ values were used in the qP_d_ equation allowing the estimation of qP_d_ in the case of a heterogeneous antenna system of *S. martensii* (qP_d het_) as described by Ware et al. (2015):

$${\mathrm{qP}}_{d\;\mathrm{het}}=\frac{F_{m}^{\prime }-F_{0\;\mathrm{act}}^{\prime }}{F_{m}^{\prime }-F_{0\;\mathrm{het}}^{\prime }}$$

pNPQ was determined according to Ruban and Murchie (2012) as the last value of NPQ corresponding to qP_d het_ > 0.98. pNPQ values relative to the independent samples were used to obtain the average pNPQ values for each plant group.

### 2.5 Light‐tolerance curves

Light tolerance curves were determined for each plant group plotting the fraction of photoinactivated samples at a given irradiance (those with qP_d het_ < 0.98) with the light intensity as described by Ware et al. (2015). Instead of the Hill equation used by Ware et al. (2015), regression curves were produced fitting the data with the following logistic equation using Origin software:

$$\text{photoinactivated samples fraction}=1-\frac{1}{1+{\left(\frac{I_{x}}{I_{50}}\right)}^{p}}$$

where I_50_ is the irradiance responsible for the photoinactivation of half the samples and I_X_ is the irradiance corresponding to a given fraction of photoinactivated samples. The fitting parameter *p* can be related to the intrinsic propensity of the specific plant group to PSII photoinactivation.

### 2.6 Data treatment

Statistical analyses and graphical representations were performed using Origin software. Statistical differences were tested by ANOVA followed by a post‐hoc Tukey test, using a threshold of *P* < 0.05.

## 3 RESULTS

### Thermal dissipation capacity in *S. martensii* acclimated to different light regimes

**FIGURE 1:**
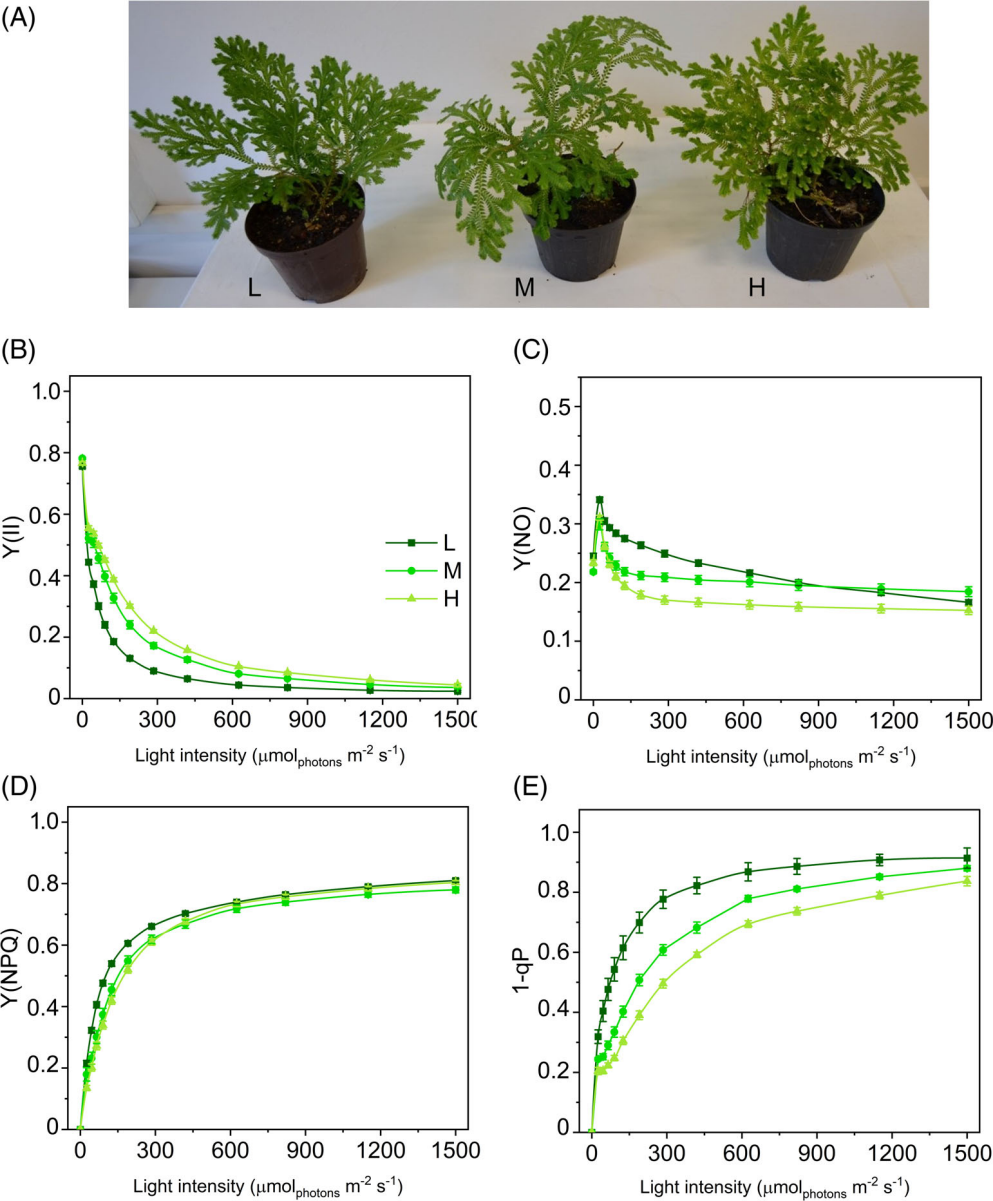
Long‐term light‐acclimation features of photosynthesis in *Selaginella martensii* acclimated to three natural light regimes. (A) Plants grown in deep shade (L, left), intermediate shade (M, center), and high light (H, right). Light curves relative to: (B) actual quantum yield of PSII photochemistry Y(II), (C) quantum yield of constitutive energy dissipation Y(NO), (D) quantum yield of regulatory thermal dissipation Y(NPQ), and (E) excitation energy pressure inside PSII (1‐qP). Average values ±standard error for *n* = 12 (L), 16 (M), 18 (H)

Compared to L and M plants, the leaf pigmentation was visibly more yellow‐green in H, as a consequence of the carotenoid accumulation, while only limited changes affected the chlorophyll *a/b* ratio (Figure 1A and Figure S1). BN‐PAGE gel analysis confirmed the low abundance of PSII‐LHCII supercomplexes, especially in H plants. The LHCII free trimers formed the most intense band in all the three plant groups without apparent differences (Figure S2; Ferroni et al., 2016). The Y(II) drop with light intensity was expectedly more marked in L plants than in M or H (Figure 1B). The higher photochemical capacity of H plants suggested a higher tolerance to PSII photoinactivation compared to the other plant groups. Such differences were more important at the early‐intermediate steps of the light curves, converging toward similarly low values at the end of the light exposure (Figure 1B). In parallel, Y(NO) was higher in L plants at low‐intermediate irradiances, confirming the lower efficiency of this plant group in light energy management (Figure 1C). The higher effectiveness in recovering Y(NO) to stable low values under increasing irradiances in H plants indicated an improved ability to control the plastoquinone pool reduction state compared with L and M plants (Tikkanen et al., 2015). This contributed to a lower excitation pressure inside PSII (1‐qP) in H plants (Figure 1E). At higher irradiances Y(NO) stabilized to a plateau value both in M and H plants, while in L plants, it decreased continuously, suggesting, in the latter, the occurrence of an additional mechanism responsible for a decrease in the electron inflow into the chain (Figure 1C). Finally, the differences in Y(NPQ) trend were relatively minor, indicating that the thermal dissipation mechanisms had a similar amplitude in the three plant groups. To the scope of the qP_d_ method, the thermal dissipation was quantified using NPQ.

**FIGURE 2:**
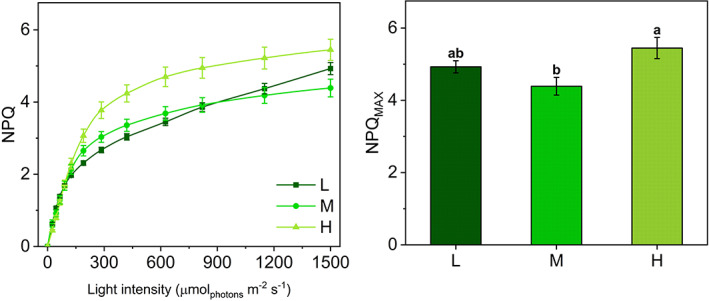
NPQ light‐response curves and maximum NPQ values in *Selaginella martensii* acclimated to three natural light regimes. (A) NPQ‐light response curves of deep shade (L, dark green), intermediate shade (M, green), and high light (H, pale green) plants during 60‐min exposure to increasing actinic light intensities. Average values ±standard error for *n* = 12 (L), 16 (M), 18 (H). (B) Maximum NPQ values (NPQ_MAX_) reached at the end of the light curve. Histogram represents average values ±standard error for *n* = 12 (L), 16 (M), 18 (H); different letters indicate a significant difference at *P* < 0.05, as determined using one‐factor ANOVA followed by Tukey's test

The steep rise in NPQ at the initial irradiances was very similar in all three groups, while the curves diverged at 125 μmol m^−2^ s^−1^ when NPQ was more intense in H plants than in M and L (Figure 2A). Such divergence was maintained between H and M plants up to the highest irradiance when it approached a plateau. Differently, in L plants NPQ increased strongly over the entire range of irradiance because of the decrease in Y(NO). The maximum capacity of thermal dissipation (NPQ_MAX_) spanned a relatively small range of values in L, M, and H plants (4.39–5.45; Figure 2B).

### 3.1 In sun plants PSII photoprotection is higher and accompanied by PSII antenna uncoupling

**FIGURE 3:**
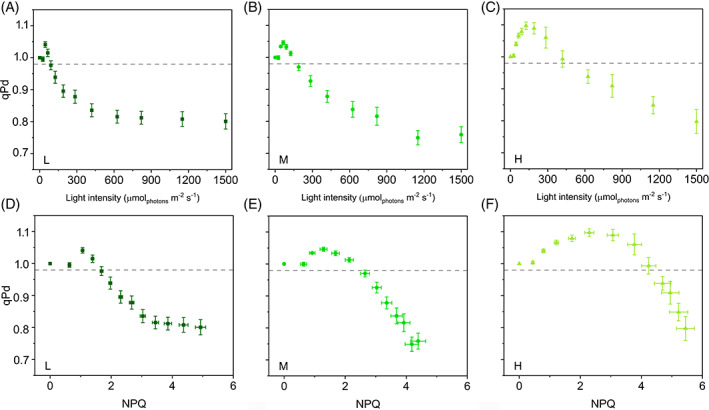
Photochemical quenching measured in the dark (qP_d_) in the hypothesis of a homogeneous antenna system in *Selaginella martensii* acclimated to three natural light regimes. (A–C) qP_d_‐light response curves of deep shade (L, dark green), intermediate shade (M, green), and high light (H, pale green) plants during 60‐min exposure to increasing actinic light intensities. (D–F) qP_d_ versus NPQ curves of L, M, and H plants. In all cases, some data points correspond to qP_d_ > 1, excluding the correctness of the homogeneous antenna model. Dashed‐gray horizontal line represents the photoinactivation threshold of qP_d_ = 0.98. Average values ± standard error for *n* = 12 (L), 16 (M), 18 (H)

Given the similar NPQ_MAX_ in all plants, we used the qP_d_ method to investigate whether the photoinhibition was likewise weakly related to the light‐acclimation regime. A drop in qP_d_ below the 0.98 thresholds indicates the onset of PSII photoinhibition and identifies at which irradiance the PSII photoprotective mechanisms start becoming less effective (Ruban & Murchie, 2012). Average qP_d_‐light response curves pointed out that the PSII sensitivity to photoinhibition could depend on the light regime (Figure 3A–C). In L plants, qP_d_ was less than 0.98 at lower irradiance (90 μmol m^−2^ s^−1^) compared to M (190 μmol m^−2^ s^−1^) and even more H (625 μmol m^−2^ s^−1^) plants. Similarly, the covariation of qP_d_ and NPQ seemed to indicate a big difference in the photoprotection offered by NPQ in H, M, and L plants, where the still photoprotective NPQ values could be around 4.7, 2.7, and 1.7, respectively (Figure 3D–F). Unfortunately, the application of the qP_d_ method in its original formulation was evidently affected by an important shortcoming since the qP_d_ values were consistently above 1 at the lower irradiances in all the plant groups. This observation strongly indicated that the two basic processes to which the qP_d_ variations are attributed (NPQ and PSII photoinactivation) were not sufficient to explain qP_d_ trends in *S. martensii*. In particular, the qP_d_ method postulates a homogeneous antenna system. However, the occurrence of PSII antenna uncoupling can produce distortions in photoinhibition monitoring using qP_d_. A modified calculation protocol allows accounting for the antenna uncoupling to obtain reliable qP_d_ in the hypothesis of antenna heterogeneity, qP_d het_ (Ware et al., 2015). According to the heterogeneous antenna model, uncoupled and loosely coupled PSII antennae are responsible for the increase in qP_d_ above 1 observed at the early stages of the light curve. This distortion is caused by an underestimation of the NPQ‐dependent F_0_′_calc_ (Oxborough & Baker, 1997) and can be compensated by applying a correction to the F_0_′_calc_ formula (see Materials and Methods for details). Such correction is exemplified for the average F_0_′_act_‐NPQ curves in Figure S3. F_0_′_act_ was fitted with a hyperbolic function of NPQ to obtain new F_0_′_calc_ values (F_0_′_het_) that be almost superimposable to F_0_′_act_ at least at the lower values of NPQ (i.e., at NPQ values corresponding to qP_d_ > 1 as in Figure 3D–F). The fitting procedure re‐established qP_d_ ca. 1 at the low irradiances and depended on three parameters: *m*, *F*, *U*.

**FIGURE 4:**
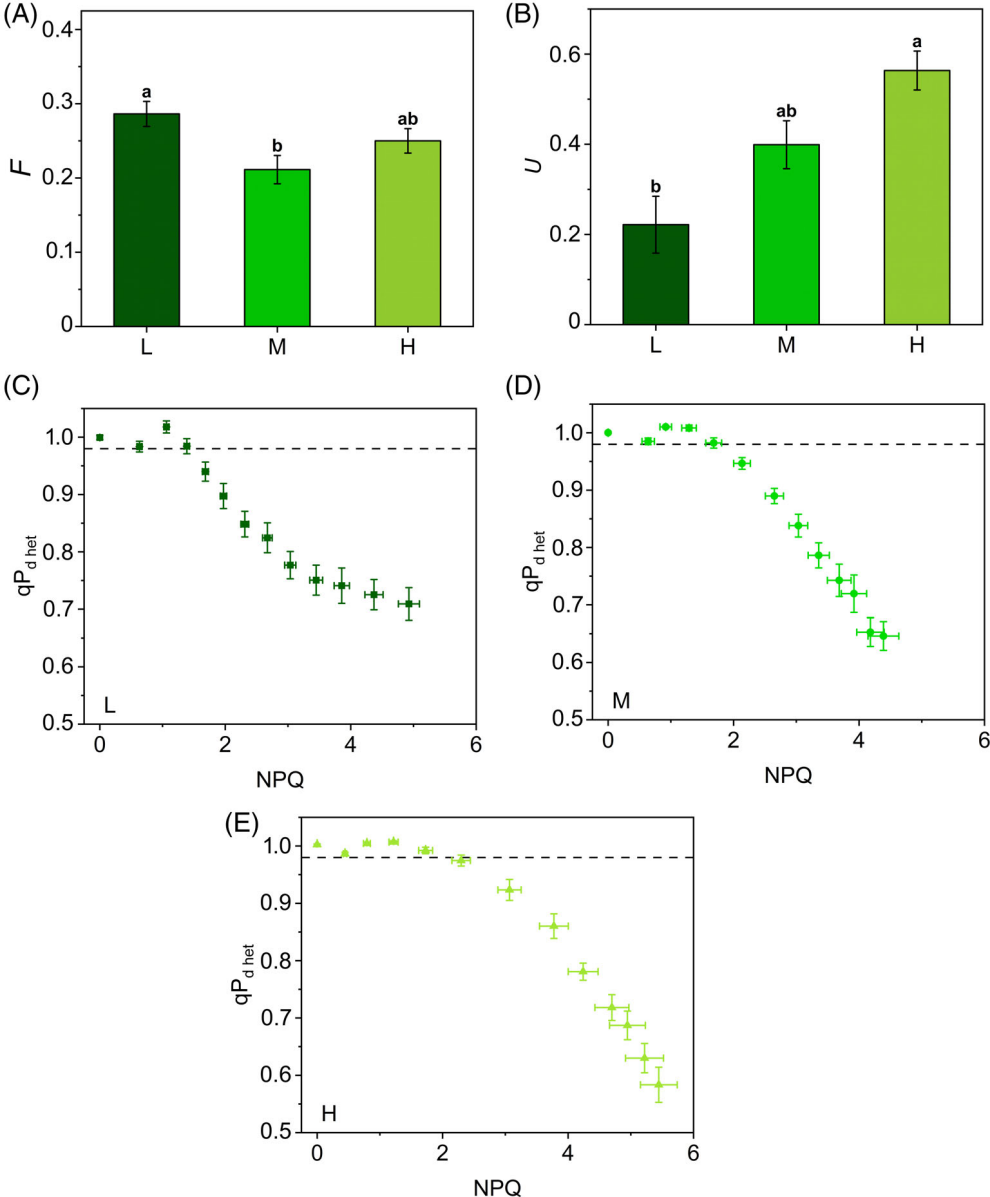
Photochemical quenching measured in the dark in context of a heterogeneous antenna system (qP_d het_) in *Selaginella martensii* acclimated to three natural light regimes. Fitting parameters (calculated as described in Material and methods): (A) the relative fluorescence F emitted by uncoupled antenna and (B) the fraction U of PSII uncoupled antenna in deep shade (L, dark green), intermediate shade (M, green) and high light (H, pale green) plants. Histograms show average values ± standard error for n = 12 (L), 16 (M), 18 (H); different letters indicate a significant difference at *P* < 0.05, as determined using one‐factor ANOVA followed by Tukey's test. (C–E) qP_d het_ versus NPQ curves of L (C), M (D) and H (E) plants during 60‐min exposure to increasing actinic light intensities. Note the effectiveness of the F and U parameters determination in reestablishing qP_d_ ≤ 1. Dashed‐gray horizontal line represents the photoinactivation threshold of qP_d het_ = 0.98. Average values ± standard error for *n* = 12 (L), 16 (M), 18 (H)

*m* and *F* represent the relative NPQ amplitude produced by uncoupled antennae and their relative fluorescence, respectively. They can be considered as “structural” parameters of the antenna system. According to Ware et al. (2015), *m* was fixed to 2, accepting estimates by Belgio et al. (2012) of a doubled quenching capacity by uncoupled antennae compared to the coupled in *Arabidopsis thaliana*. Ware et al. (2015) assumed a constant *F* equal to 0.5. Differently, we let *F* vary freely between 0 and 1: *F* was quite uniform in the three plant groups, fluctuating between 0.21 and 0.29 (Figure 4A). These lower values may indicate a structurally lower fluorescence emission by uncoupled antennae in the phylogenetically distant *S. martensii*. However, the correctness of *m* and *F* strongly depends on the validity of the assumption of the heterogeneous antenna model proposed by Ware et al. (2015). For instance, in an artificial system, the quenching capacity by uncoupled antennae was found to be similar or even smaller than that of the coupled antennae (Tian et al., 2015). However, in our study, fitting tests using different combinations of *F* and *m*, for example, closer to the value of 1, were unproductive. Conversely, the Ware's heterogeneous antenna model is internally consistent and indicated that the apparent differences among plant groups were almost exclusively due to the parameter *U*. This is the fraction of uncoupled antenna and can vary between 0 (all antenna is coupled with PSII) and 1 (all antenna is uncoupled from PSII). L plants had *U* = 0.22, a value close to what estimated by Ware et al. (2015) in *A. thaliana* grown under low light (0.15–0.20; Figure 4B). In striking contrast, the M and H samples uncoupled ca. 2 and 3 times more antenna, respectively, reaching in the latter an outstanding level of PSII antenna uncoupling (Figure 4B).

**FIGURE 5:**
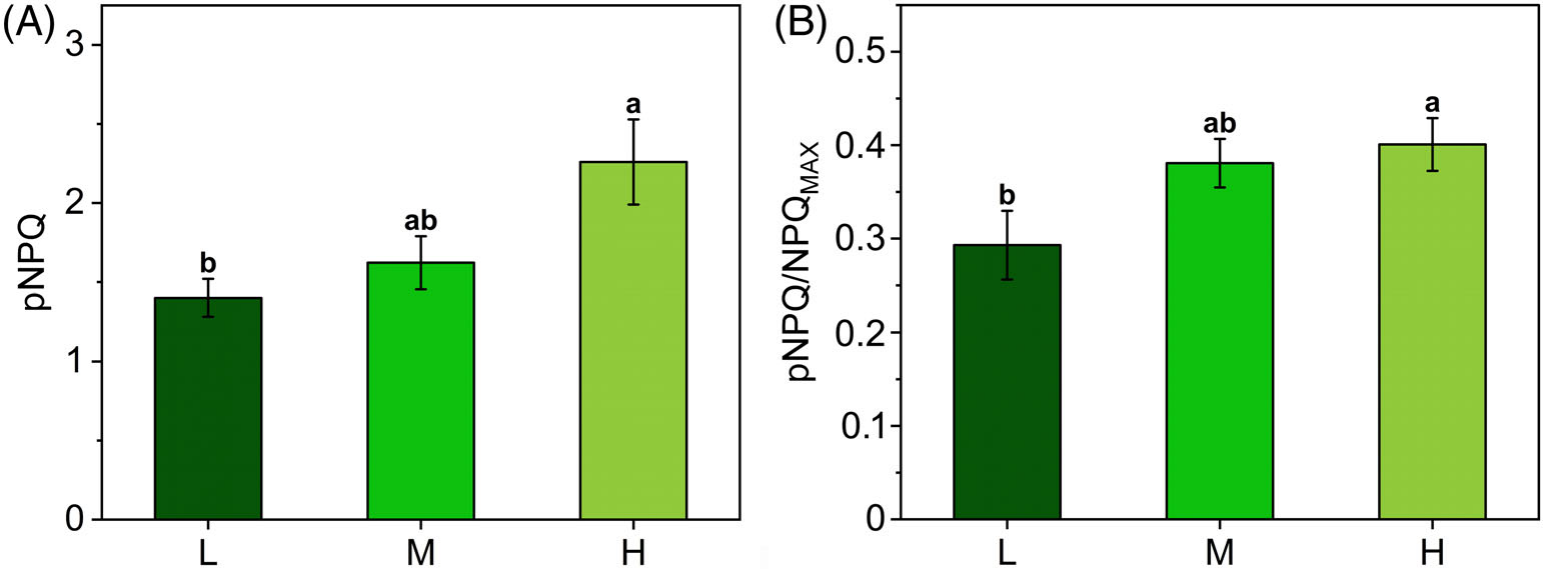
Quantification of photoprotection offered by the non‐photochemical quenching (NPQ) in *Selaginella martensii* acclimated to deep shade (L, dark green), intermediate shade (M, green), and high light (H, pale green). (A) Photoprotective NPQ (pNPQ) in L, M, and H plants calculated following qP_d het_ versus NPQ curves analysis as in Figure 4. (B) Ratio between pNPQ and maximum NPQ (NPQ_MAX_). Histograms represent average values ± standard error for *n* = 12 (L), 16 (M), and 18 (H); different letters indicate a significant difference at *P* < 0.05, as determined using one‐factor ANOVA followed by Tukey's test

qP_d_ values were corrected replacing F_0_′_calc_ with F_0_′_het_ in the qP_d_ equation, leading to the new parameter qP_d het_. qP_d het_ did not increase above 1 during the early steps of the light routine and was suitable for the quantification of the photoprotection offered by NPQ during 55 min of increasing actinic light illumination. pNPQ is now defined as the last value of NPQ that maintains qP_d het_ above the 0.98 threshold (Ware et al., 2015). qP_d het_ was maintained above 0.98 for higher NPQ values in H plants than in M and L, showing a higher photoprotective capacity in the former, although to a well‐reduced extent as compared to the estimates from uncorrected qP_d_ (see Figure 3A–C and 4C–E for comparison). H plants benefitted from 29% and 38% more pNPQ than M and L, respectively (Figure 5A). Photoinhibitory irradiances were re‐determined as 70, 90, and 120 μmol m^−2^ s^−1^ for L, M, and H, respectively. Although pNPQ estimation can be strongly dependent on the light treatment (length, number, and intensity of the light intervals), pNPQ was strongly consistent with the growth light regime, in contrast to what observed for NPQ_MAX_ (Figure 2B). The pNPQ/NPQ_MAX_ ratio reported in Figure 5B indicated that ca. 40% of NPQ_MAX_ was photoprotective in H and M plants, while in L plants the photoprotective fraction was reduced to 29%.

### 3.2 Validation of qP_d_ sensitivity to the onset of PSII photoinhibition

The light curves account for cumulative light‐related effects occurring on the same sample exposed to increasing light intensities, but cannot allow a direct comparison of qP_d_ and PSII photoinhibition. To this aim, the qP_d_ method was also applied on the data obtained after independent light inductions to stable irradiances, followed by dark relaxation (see induction curves of NPQ, Figure S4A–J).

To allow an easier comparison of results obtained with two protocols (light curve or individual inductions), the qP_d_ values were sampled during the light induction based on the total number of photons conveyed to the sample. For instance, at the end of the step of 190 μmol m^−2^ s^−1^ in the light curve, 2700 μmol m^−2^ photons had been directed to the sample; during the induction curve at 190 μmol m^−2^ s^−1^, a very close number of photons (2660 μmol m^−2^) reached the sample after 14 min. The qP_d_ trends obtained from the induction curves were overall consistent with those derived from the light curves, including qP_d_ > 1 at low‐medium irradiance (Figure S5 and Figure 5A–C). Similar to the original light‐curve‐based method, the actual level of PSII photoinhibition remained unknown.

**FIGURE 6:**
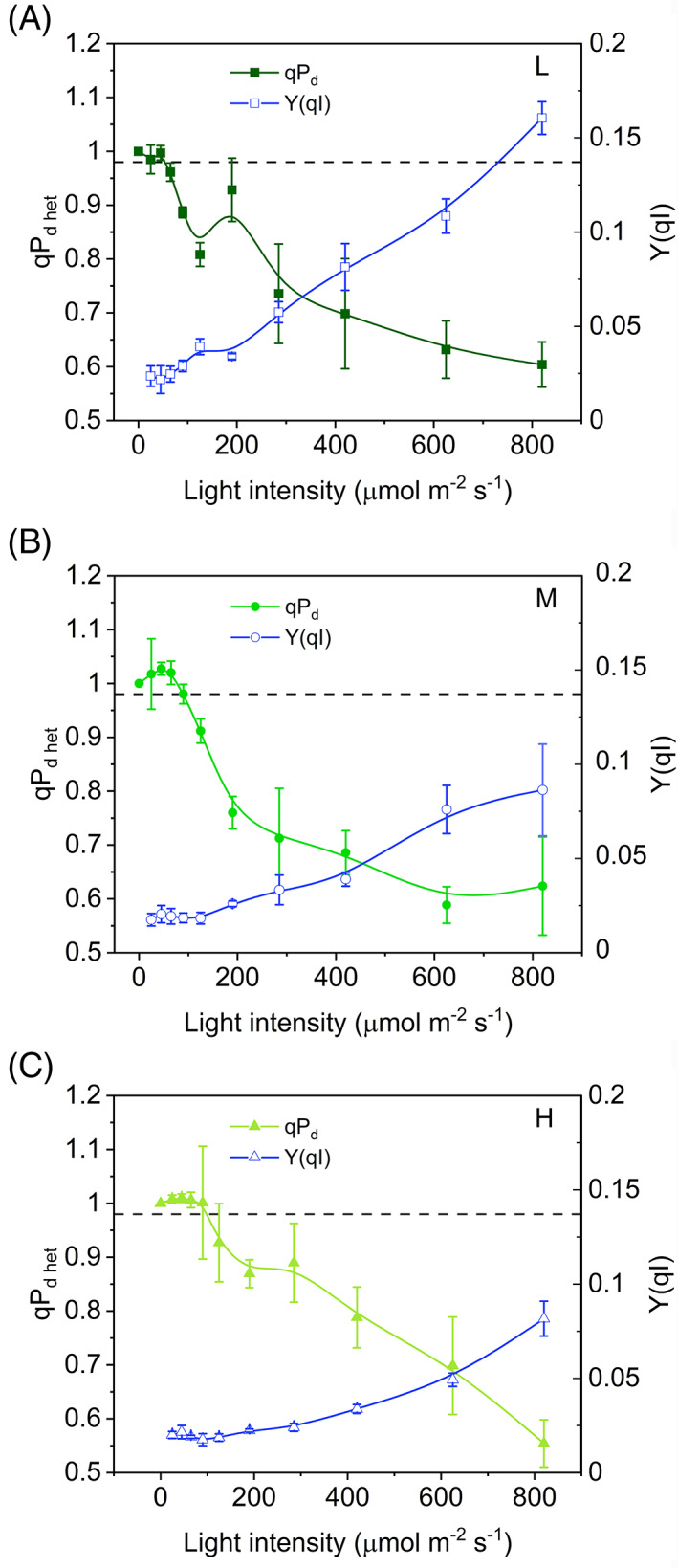
Comparison between photochemical quenching measured in the dark in context of a heterogeneous antenna system (qP_d het_) and quantum yield of PSII photoinhibition (Y(qI)) as functions of the light intensity in *Selaginella martensii* acclimated to three natural light regimes—deep shade (A), intermediate shade (B) and high light (C) (see the text for details). In all plants, stable and low values of Y(qI) correspond to stable qP_d het_ around 1. The increment in Y(qI) corresponds to a drop in qP_d het_. Average values ± standard error for *n* = 3–5

Because the link between qP_d_ and photoinhibition is of vital importance to a well‐grounded use of qP_d_ in *S. martensii*, qP_d het_ values were then calculated at the end of the induction curves to compare them with the recovered F_v_/F_m_ after the dark relaxation. The lost PSII yield is photoinhibition per definition, Y(qI). The preliminary fitting procedure of F_0_′_act_ values allowed the estimation of increasing *U* from L to M and H plants (0.39, 0.61, and 0.82). Higher *U* values compared to the light‐curve experiment could be due to the different protocol used and the longer natural photoperiod experienced by the plants in the greenhouse (14 vs. 9 h day^−1^). The resulting qP_d het_‐light curves revealed that, for stable and low values of Y(qI) (0.02), qP_d het_ remained constantly and consistently above 0.98 (Figure 6A–C). Subsequently, when Y(qI) started to increase, qP_d het_ dropped below 0.98, but, as expected, at lower light intensities in L plants compared to M and H (approximately 65, 125, and 125 μmol m^−2^ s^−1^, respectively, Figure 6A–C). Therefore, despite the strong assumptions in the interpretation of qP_d_, even stronger in context of antenna heterogeneity, we obtained empirical evidence of the link between qP_d het_ decay and the onset of PSII photoinhibition in *S. martensii*. Importantly, qP_d het_ < 0.98 characterized the absence of photoinhibition, that is, the effectiveness of photoprotection. Again, higher levels of photoprotection were linked to higher values of *U*. Notwithstanding the different experimental setup, the irradiances determining the start of photoinhibition were close to those obtained in the original protocol of the qP_d het_ experiment.

### 3.3 qP_d_ _het_ decreasing phase under high light reveals a surprisingly strict control of photoinhibition in L plants

The second phase of the qP_d het_ curves (qP_d het_ < 0.98) was characterized by a monotonous decay of qP_d het_, which generally does not add further information about PSII photoprotection, as confirmed in M and H *S. martensii* plants (Figure 4D–E). Surprisingly, qP_d het_ decay in L plants tended instead to stabilize at the highest irradiances, remarkably diverging from the simpler trends observed in M and H, and resulting in higher qP_d het_ final values (Figure 4C), that is, a mitigation of the PSII photoinhibition rate had occurred at the end of the light routine. The stabilization of qP_d het_ values might be credited to the NPQ action. The slowing down the qP_d het_ decay in L plants was due to the linear decrease in F_0_′_act_ as a function of NPQ (Figure S3). In particular, the quenching of F_0_′_act_ can be assigned to the marked NPQ increase occurring during the late stages of the light curve, characterizing specifically the L plants (Figure 2A). This result shows that enhanced thermal dissipation processes could effectively contribute to mitigate PSII photoinhibition rate in L plants at irradiance levels > 400 μmol m^−2^ s^−1^ (Figure 4C–E).

### 3.4 Light tolerance curves offer an alternative and consistent quantification of phototolerance

**FIGURE 7:**
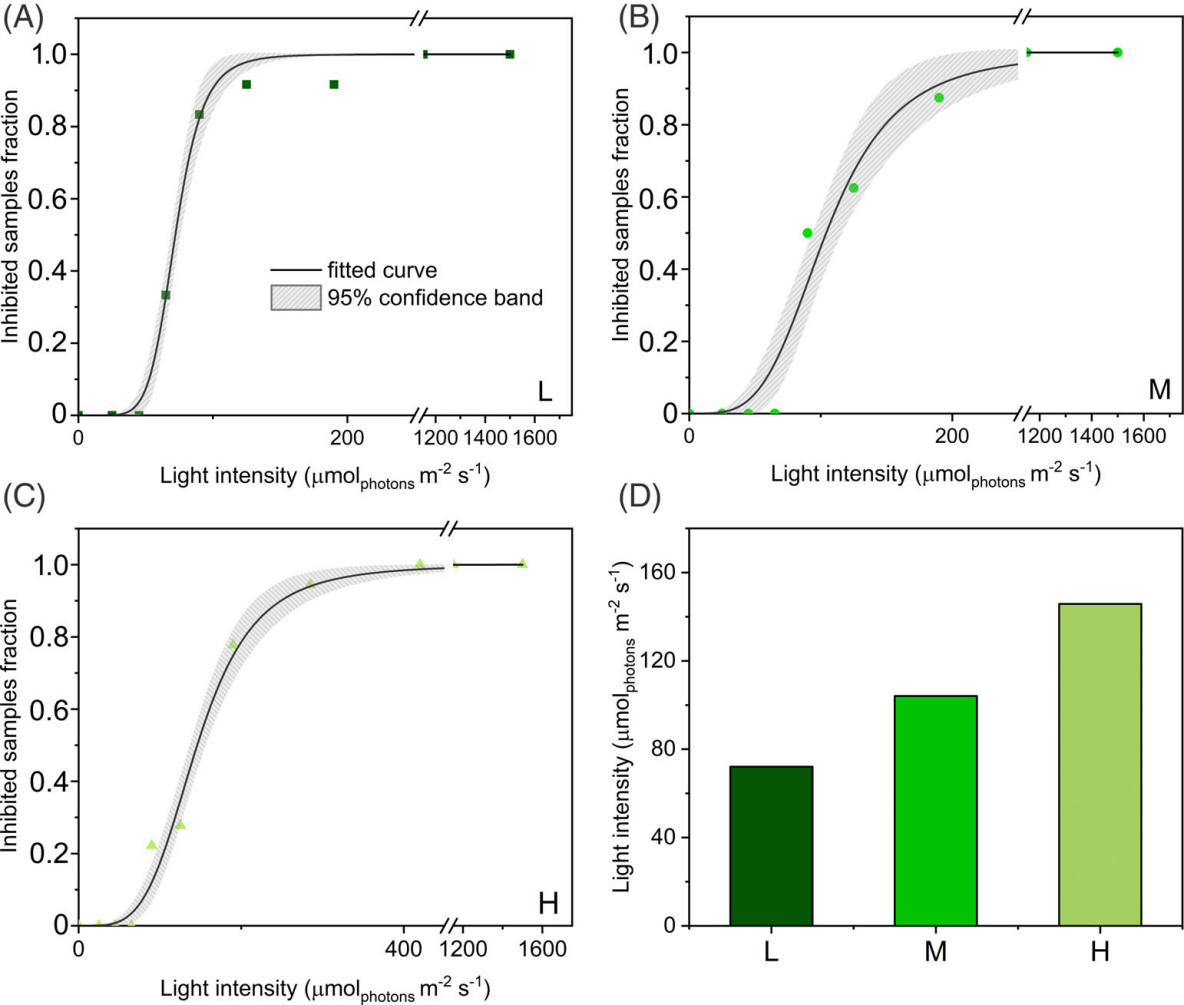
Tolerance to photoinhibition in *Selaginella martensii* acclimated to different natural light regimes. (A–C) Light tolerance curves of plants acclimated to deep shade (L, dark green), intermediate shade (M, green) and high light (H, pale green); fitting curves (black lines) were obtained with the logistic equation and are reported with 95% confidence bands. (D) Values of half‐inhibiting samples irradiance (I_50_) obtained with the logistic fitting

To further substantiate the results of comparative photoprotection in *S. martensii* plants, light tolerance curves were built on the same datasets and used as an approach independent from the pNPQ quantification. Plots of light intensity against the respective fraction of photoinactivated samples (those yielding qP_d het_ < 0.98)were fitted with a logistic function: an increased steepness of the curve indicates a higher propension of plants to PSII photoinactivation. Phototolerance was estimated by I_50_ parameter, that is, the light intensity causing the PSII inactivation in half the analyzed samples. The sensitivity of PSII to photoinactivation decreased from shade to sun acclimation (Figure 7A–C). However, despite the strongly contrasting light regimes, the difference in I_50_ between the two extremes was of only 74 μmol m^−2^ s^−1^ (72 vs. 146 μmol m^−2^ s^−1^ in L and H plants, respectively; Figure 7D).

**FIGURE 8:**
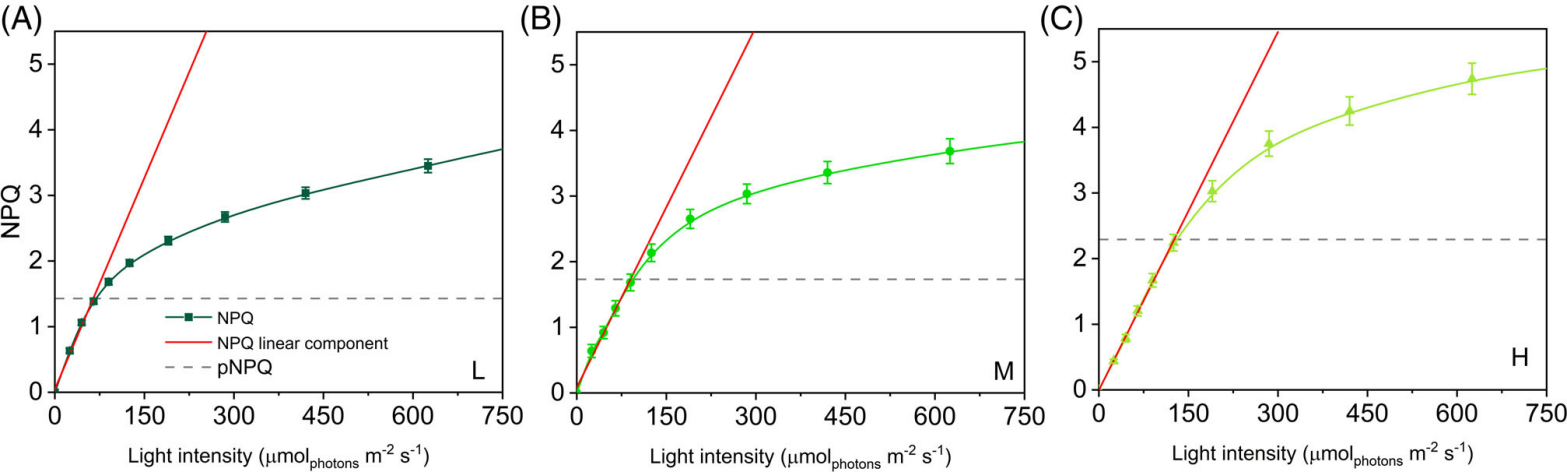
Light‐tolerance and photoprotective NPQ (pNPQ) in *Selaginella martensii* acclimated to different natural light regimes. Linear fitting (red lines) relative to the first, steepest increasing phase of NPQ‐light curves (green lines) in L (A), M (B), and H (C) plants. Gray‐dashed horizontal lines indicate the photoprotection offered by thermal dissipation to PSII (pNPQ) in each plant group (see Figure 5). NPQ loses its linear response to light at NPQ > pNPQ. Each point represents average value ± standard error for *n* = 12 (L), 16 (M), and 18 (H)

Phototolerance trends revealed by I_50_ resembled the gradient previously observed for pNPQ (Figures 5A and 7D). Because there was no obvious relationship between pNPQ and NPQ_MAX_, it was interesting to find out how the I_50_ and pNPQ positioned on the NPQ‐light curves. For each type of long‐term acclimation, the position of pNPQ marked the end of the linear growth of NPQ in response to increasing irradiance; for NPQ > pNPQ (or irradiance>I_50_) the linear response with light intensity was lost, that is, NPQ increased more slowly (Figure 8A–C). This scenario was uniform in all the analyzed samples and indicated that PSII photoprotection was efficient until NPQ increased linearly as a function of light intensity.

## 4 DISCUSSION

The present study demonstrates that in the ancient vascular plant *S. martensii* the pNPQ is not proportional to the total NPQ amplitude (NPQ_MAX_) inducible in plants acclimated to strongly contrasting light regimes. Instead, the PSII photoprotection effectiveness is strongly dependent on the light regime, with a remarkable increase in pNPQ from L to H plants (Figure 5A). Developmental acclimation to higher light availability results indeed in a higher phototolerance to increasing irradiance (Figure 7). The inconsistency between pNPQ and NPQ_MAX_ finds its major explanation in the special regulation of excitation energy management in deep‐shade plants when exposed to exceedingly high light.

According to Ruban (2016), pNPQ is mainly due to qE. Because in angiosperms qE is more induced in sun‐grown plants, it can be satisfying to explain the variations in photoprotective capacity upon long‐term light‐acclimation (Anderson & Aro, 1994; Demmig‐Adams et al., 2015; Mathur et al., 2018; Mishra et al., 2012; Park II et al., 1997; Schumann et al., 2017). Differently, in *S. martensii*, qE is only slightly variable between L, M, and H plants (Ferroni et al., 2016). A different view about the PSII photoprotection offered by NPQ was presented by Lambrev et al. (2012), based on ultrafast time‐resolved fluorescence measurement in *A. thaliana*. Although qE contributes largely to the total NPQ amplitude, it was not considered the main component of photoprotective NPQ, but qZ was instead proposed as the prevailing mechanism that brings photoprotection to PSII (Lambrev et al., 2012). However, the interpretation of the same kinetic NPQ component as qZ in *S. martensii* is quite questionable (Ferroni, Colpo, et al., 2021). In fact, rather than depending on zeaxanthin, this component, termed qX, seems to be triggered by a reduced electron transport chain and to exploit PSI as a thermal quencher to prevent PSII photodamage (Ferroni et al., 2018; Ferroni, Colpo, et al., 2021). qX activity is deemed related to PSII interactions with PSI as mediated by LHCII (Ferroni et al., 2014), not only the formation of the state transition complex (Galka et al., 2012; Pesaresi et al., 2009; Wood & Johnson, 2020), but also the assembly of PSI‐LHCII‐PSII megacomplexes responsible for an extensive connectivity between photosystems, including the chance for energy spillover of excitation energy from PSII to PSI (Barber, 1980; Grieco et al., 2015; Jajoo et al., 2014; Järvi et al., 2011; Tiwari et al., 2016; Yokono et al., 2015). Currently, energy spillover in megacomplexes is considered relevant to effective PSII photoprotection (Yokono et al., 2019).

Because the gradient in pNPQ cannot be explained by variations of qE/qZ in *S. martensii*, alternative explanations could be related to other regulatory functions of the antenna system. According to the qP_d_ method, pNPQ determination is based on the comparison between the ideal F_0_′_calc_ and the measured F_0_′_act_, leading to qP_d_ values lower than 1 as a mark for photoinhibition onset. More problematic are qP_d_ values above 1. Any distortion in F_0_′_act_ could be in principle due to the PSII photoinhibition: in fact, only F_0_′_act_ determination is affected by PSII photoinhibition, while F_0_′_calc_ should be insensitive (Oxborough & Baker, 1997). If this were the case, we should observe a rise in the measured values of F_0_′_act_ compared to the calculated, while the results show a completely opposite scenario in which F_0_′_act_ is lower than F_0_′_calc_. Moreover, negligible values of Y(qI) during the qP_d_ rise are a straightforward demonstration that qP_d_ > 1 cannot be due to photoinhibition. Such lower‐than‐expected F_0_′_act_ suggests instead the occurrence of quenching mechanisms of F_0_′ in addition to the direct effect of NPQ. According to the interpretation of average quenching properties of uncoupled antennae offered by Belgio et al. (2014) and Ware et al. (2015), the role of additional F_0_′ quenchers could be played by the antennae uncoupled from PSII. These hypothetical quenchers would be characterized by an enhanced NPQ capacity and by a lower fluorescence emission than the coupled population. At present, this is the only well‐modeled interpretation of qPd > 1 and, as such, it was used in our work. Accordingly, *S. martensii* would be characterized by a larger population of uncoupled/loosely coupled antenna than the angiosperms, in particular *A. thaliana* (Ware et al., 2015). In the latter, the antenna uncoupling distorting the qP_d_ trends is specific to the low light‐grown plants and related to the acclimative accumulation of LHCII (Ware et al., 2015). It is not surprising that the shade‐tolerant lycophyte *S. martensii* is affected by similar distortions, because of the great amount of LHCII as compared to PSII (Ferroni et al., 2014, 2016). However, with respect to the developmental acclimation to light, *S. martensii* behaves exactly the opposite of *A. thaliana*: *U* markedly increases from L to H plants, suggesting a massive use of LHCII antenna uncoupling. In H plants, the invariant quantity of free LHCII trimers becomes accordingly overabundant in comparison with the reduced amount of PSII reaction centers (Ferroni et al., 2016). Therefore, in *S. martensii* the involvement of uncoupled quenched antennae in qP_d_ determination seems well grounded from a biochemical point of view. However, considering that the excitation quenching capacity by uncoupled LHCII is a debated issue, other explanations are possible (Tian et al., 2015). Another reason for a too low F_0_′_act_ at non‐photoinhibitory irradiances could be the reduction in PSII absorption cross‐section due to state‐transition‐like antenna detachment. Interestingly, in M plants the maximum divergence between F_0_′_act_ and F_0_′_calc_—that is, the peak in qP_d_—is in very good agreement with the peak of LHCII phosphorylation previously reported (Ferroni et al., 2018). Both events occur approximately at the irradiance of growth (50–100 μmol m^−2^ s^−1^). If the qP_d_ increase is a reflection of state‐transition‐like processes, the antenna uncoupling plays again a pivotal role. This inference allows the interpretation of qP_d_ in the more general frame of the multiple roles assigned to the free LHCII in the thylakoid membrane, including the regulation of PSI‐PSII interaction at the grana margins (Grieco et al., 2015; Wientjes et al., 2013; Wood & Johnson, 2020; Zivcak et al., 2014). It is very probable that a more complete interpretation of qP_d_ should also take into account the photoprotective contribution by PSI, together with mixed populations of uncoupled LHCII, which could be “functionally isolated” from PSII (quenched or unquenched) and/or connected to PSI.

Among PSII uncoupled antennae, a consistent fraction probably serves as qE quenching site (Holzwarth et al., 2009; Miloslavina et al., 2011; Ruban, 2016). Because in *S. martensii* the qE amplitude is almost invariable irrespective of the light regime (Ferroni et al., 2016), the remaining, non‐qE‐related fraction of uncoupled antenna must be responsible for the increased photoprotection from L to H plants, for example, via interactions involving PSI as a photochemical or non‐photochemical quencher (Brestic et al., 2015; Tiwari et al., 2016; Wood & Johnson, 2020; Yokono et al., 2019). In *S. martensii* the amount of PSI and PSI‐LHCII‐PSII megacomplexes increases under high light (Ferroni et al., 2016) and the assembly of the latter requires the recruitment of free LHCII trimers to mediate labile interactions between PSII and PSI (Grieco et al., 2015). Terashima et al. (2021) suggested that the energy spillover process could be particularly important in shade‐tolerant plants to confer them resistance against strong sunflecks. In a lycophyte with invariable LHCII amount and low carbon fixation capacity (Ferroni et al., 2016; Ferroni, Brestič, et al., 2021), the extensive PSII antenna uncoupling can allow an emphasized exploitation of PSI‐linked photoprotection also upon long‐term acclimation to high light. Conversely, in the complete absence of sunflecks, the photoprotective role of uncoupled antennae and PSI seems diminished, potentially exposing the small PSI pool of L plants to photodamage upon short‐term exposure to even moderate light (Brestic et al., 2015). Because PSI is particularly sensitive to donor‐side over‐reduction (Takagi et al., 2017), its photoprotection primarily depends on a reduced inflow of electrons from PSII into the membrane (Yamamoto & Shikanai, 2019). The qP_d het_ results suggest that in L plants of *S. martensii* the safe accumulation of a stable population of photoinactivated PSII under moderate/high light may serve to the scope of downregulating the electron flow and preserve PSI (Tikkanen et al., 2015). Beside photoprotective thermal dissipation mechanisms, PSII photoinactivation is also counteracted by the repair cycle of PSII based on the D1 core protein turnover (Keren et al., 1995, Baena–González & Aro, 2002, Kato & Sakamoto, 2009, Nath et al., 2013). The PSII repair cycle requires the migration of photodamaged PSII to the non‐appressed grana margins, where the turnover takes place (Anderson & Aro, 1994; Järvi et al., 2015; Li et al., 2018; Kirchhoff, 2014). D1 turnover is more active in sun plants, whose thylakoid membranes are enriched in grana margins (Anderson & Aro, 1994). Differently, shade plants are characterized by a higher grana stacking, further increased when exposed to high irradiance; the extensive thylakoid appression hinders the PSII turnover, so that the grana contain a kind of reservoir of inactive PSII (Anderson & Aro, 1994; Mathur et al., 2018; Matsubara & Chow, 2004; Tietz et al., 2015). The accumulation of photoinactivated PSII upon increasing irradiances also occurs in *S. martensii*, starting from relatively low light intensities (see I_50_ values, Figure 7D). However, in L plants—the richest in PSII—qP_d_ surprisingly slows its drop at the highest irradiances, indicating the achievement of a constant ratio between intact and photoinactivated PSII (Figure 4C). A stable reservoir of photoinactivated PSII in long appressed pseudo‐lamellar thylakoids may have an important photoprotective role, because they safely dissipate the excess of absorbed energy, preventing the photoinactivation of the remaining, active PSII, but also restricting the electron inflow directed to PSI (Mathur et al., 2018; Matsubara & Chow, 2004). According to Ruban (2016), qI does not contribute to pNPQ. However, the qP_d_ method indirectly evidences the physiological function of qI in mitigating the PSII photoinactivation, although the small qI extent (5%–10% of total NPQ amplitude, Ferroni et al., 2016) could not explain per se the constant increase in NPQ at high irradiances observed in L plants (Figure 2A). A possible interpretation of this phenomenon can be related to an additional thermal dissipation mechanism produced by PSII cores (Nicol et al., 2019), more relevant in L plants because of their higher content in PSII.

In conclusion, although qE might still represent the main component of pNPQ as postulated by Ruban (2016), in *S. martensii* the pNPQ could also strongly depend on the PSII antenna uncoupling and the relative amount of PSII and PSI. After the correction for the antenna heterogeneity, qP_d het_ is confirmed as a very precise indicator of incipient PSII photoinhibition. Furthermore, the example of *S. martensii* suggests that the qP_d_ method can be sensitive to PSI‐related mechanisms and to the PSII core‐related thermal dissipation. A sustained PSII photoinhibition can have a photoprotective function to increase physiologically a low PSI/PSII ratio (Shimakawa & Miyake, 2019). Evidence for the importance of such processes is quite sparse in the literature regarding angiosperms. The results obtained in *S. martensii* may indicate that processes collateral to qE, and often considered as minor, can have had a special relevance for thylakoid membrane photoprotection in ancient land plants, which do not modulate extensively the LHCII total content (Gerotto et al., 2019; Lei et al., 2021). However, any evolutionary conclusion should be cautious taking into consideration millions of years of parallel evolution of *Selaginella*, making it difficult to define a certain physiological trait as primitive or derived. For instance, some properties evidenced in *S. martensii* could be shared by other shade plants because of convergent evolution. This study invites the validation (or not) of the qP_d_ method and its conclusions emerging in a lycophyte in other plants sharing the same deep‐shade habitat and long‐term invariable LHCII amount.

## AUTHOR CONTRIBUTIONS

Lorenzo Ferroni conceived and supervised the experiment; Andrea Colpo planned and performed the experiments; Andrea Colpo and Alessandra Sabia performed the data analysis; Andrea Colpo, Costanza Baldisserotto, Simonetta Pancaldi, Lorenzo Ferroni analyzed and interpreted the results; Andrea Colpo and Lorenzo Ferroni wrote the manuscript; all authors edited the manuscript.

## Supporting information


**Figure S1.** Photosynthetic pigments quantification in *Selaginella martensii* acclimated to deep shade (L), intermediate shade (M) and full sunlight (H).
**Figure S2**. Native thylakoid composition in *Selaginella martensii* acclimated to deep shade (L), intermediate shade (M) and full sunlight (H).
**Figure S3**. Examples of minimum fluorescence as a function of Non‐Photochemical Quenching (NPQ) in *Selaginella martensii*.
**Figure S4**. NPQ kinetics curves of *S. martensii* plants recorded at different light intensities.
**Figure S5**. Photochemical quenching measured in the dark in *S. martensii* plants acclimated to deep shade (L), intermediate shade (M) and full sunlight (H) upon independent exposure to increasing irradiances.

## Data Availability

The data supporting the findings of this study are available from the corresponding author Lorenzo Ferroni, upon request.
