# Supplementary material for: Photosystem II photoinhibition and photoprotection in a lycophyte, Selaginella martensii
Source: Physiol Plant. 2021 Dec 6;174(1):e13604. doi: 10.1111/ppl.13604 (PMC9300044; doi:10.1111/ppl.13604)
Supplement: Supplementary file 1 — Figure S1. Photosynthetic pigments quantification in Selaginella martensii acclimated to deep shade (L), intermediate shade (M) and full sunlight (H). Figure S2. Native thylakoid composition in Selaginella martensii acclimated to deep shade (L), intermediate shade (M) and full sunlight (H). Figure S3. Examples of minimum fluorescence as a function of Non‐Photochemical Quenching (NPQ) in Selaginella martensii. Figure S4. NPQ kinetics curves of S. martensii plants recorded at different light intensities. Figure S5. Photochemical quenching measured in the dark in S. martensii plants acclimated to deep shade (L), intermediate shade (M) and full sunlight (H) upon independent exposure to increasing irradiances. [file PPL-174-0-s001.docx]

**Photosystem II photoinhibition and photoprotection in a lycophyte, *Selaginella martensii***

Andrea Colpo, Costanza Baldisserotto, Simonetta Pancaldi, Alessandra Sabia, Lorenzo Ferroni

Department of Environmental and Prevention Sciences, University of Ferrara, Corso Ercole I d’Este 32, 44121 Ferrara, Italy.

Supporting information

**Fig. S1.** Photosynthetic pigments quantification in *Selaginella martensii* acclimated to deep shade (L), intermediate shade (M) and full sunlight (H).

**Fig. S2.** Native thylakoid composition in *Selaginella martensii* acclimated to deep shade (L), intermediate shade (M) and full sunlight (H).

**Fig. S3.** Examples of minimum fluorescence as a function of Non-Photochemical Quenching (NPQ) in *Selaginella martensii.*

**Fig. S4.** NPQ kinetics curves of *S. martensii* plants recorded at different light intensities.

**Fig.S5.** Photochemical quenching measured in the dark in *S. martensii* plants acclimated to deep shade (L), intermediate shade (M) and full sunlight (H) upon independent exposure to increasing irradiances.


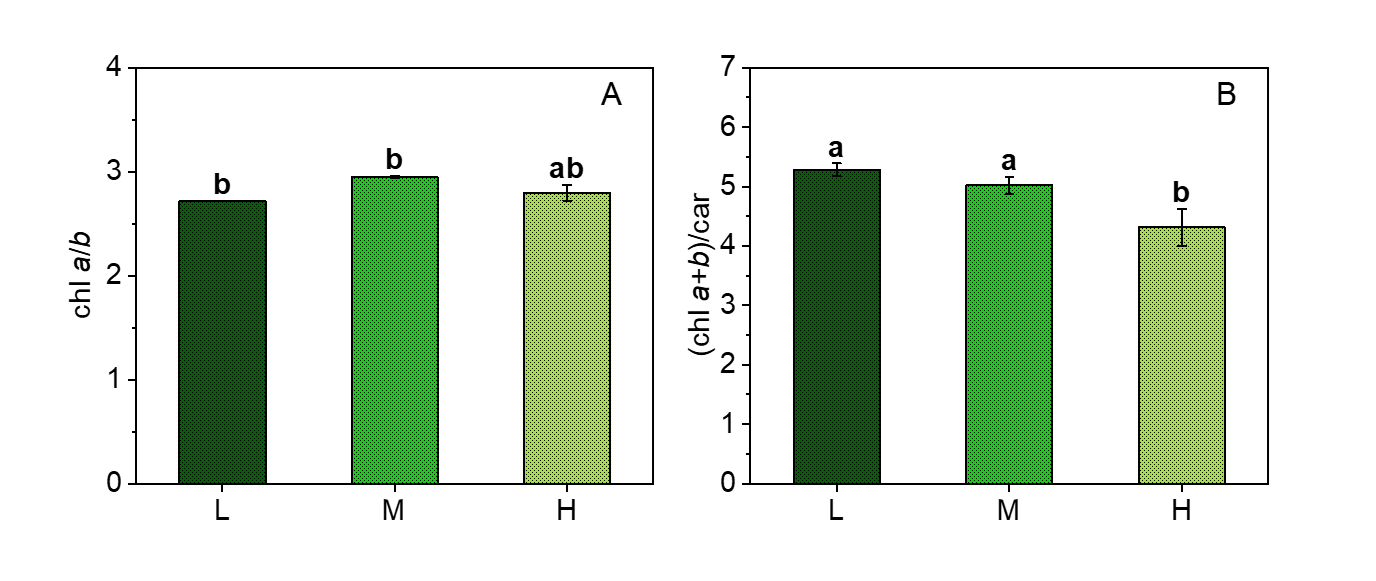


**Fig. S1**. Photosynthetic pigments quantification in Selaginella martensii acclimated to deep shade (L), intermediate shade (M) and full sunlight (H). Pigment ratios in isolated thylakoids are reported as the molar ratio between chlorophyll (chl) a and b content (A) and chlorophyll a+b and carotenoids (B). Histograms represent average values ±Standard Error for n=3. Different letters indicate a significant difference determined with ANOVA followed by Tukey’s test at p<0.05.


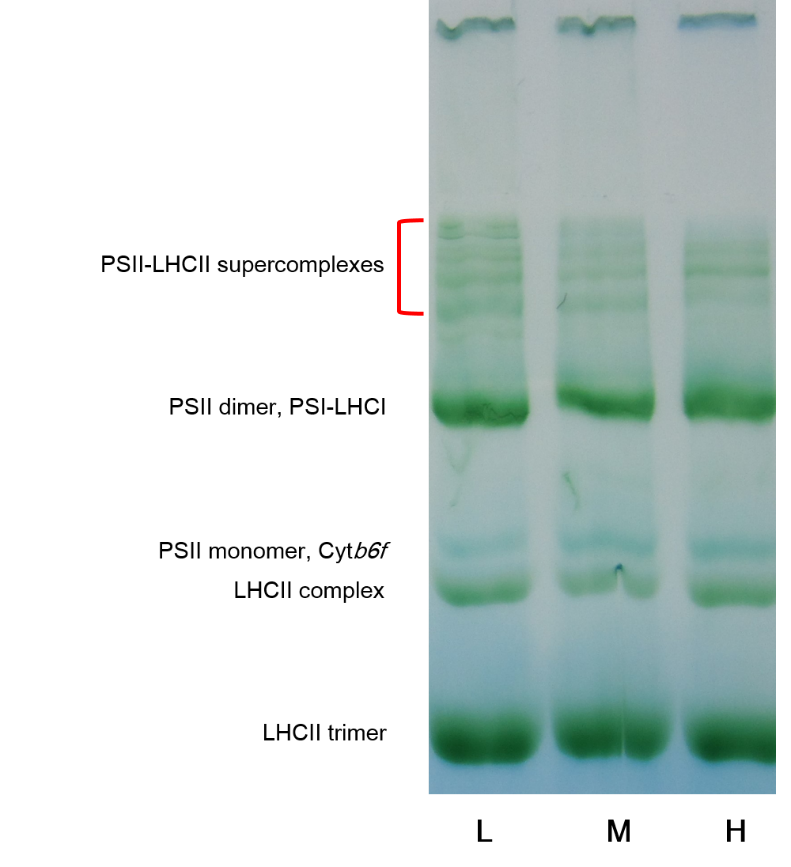


**Fig. S2**. Native thylakoid composition in Selaginella martensii acclimated to deep shade (L), intermediate shade (M) and full sunlight (H). Thylakoids corresponding to 8 mg of chlorophyll were solubilized with 1.5% β-dodecylmaltoside to a final chlorophyll concentration of 0.5 mg ml^-1^ and subjected to Blue-Native Polyacrylamide gel Electrophoresis (BN-PAGE). Electrophoretic bands were identified according to Järvi et al. (2011) and Ferroni et al. (2014). The heavy PSII-LHCII supercomplexes are more abundant in L plants than in M or H plants. In contrast, the intensity of the band corresponding to the LHCII trimers is almost invariable in the three types of plants. The thylakoid organization in the plant material used for the fluorometric experiments is therefore uniform to that used in Ferroni et al. (2016).


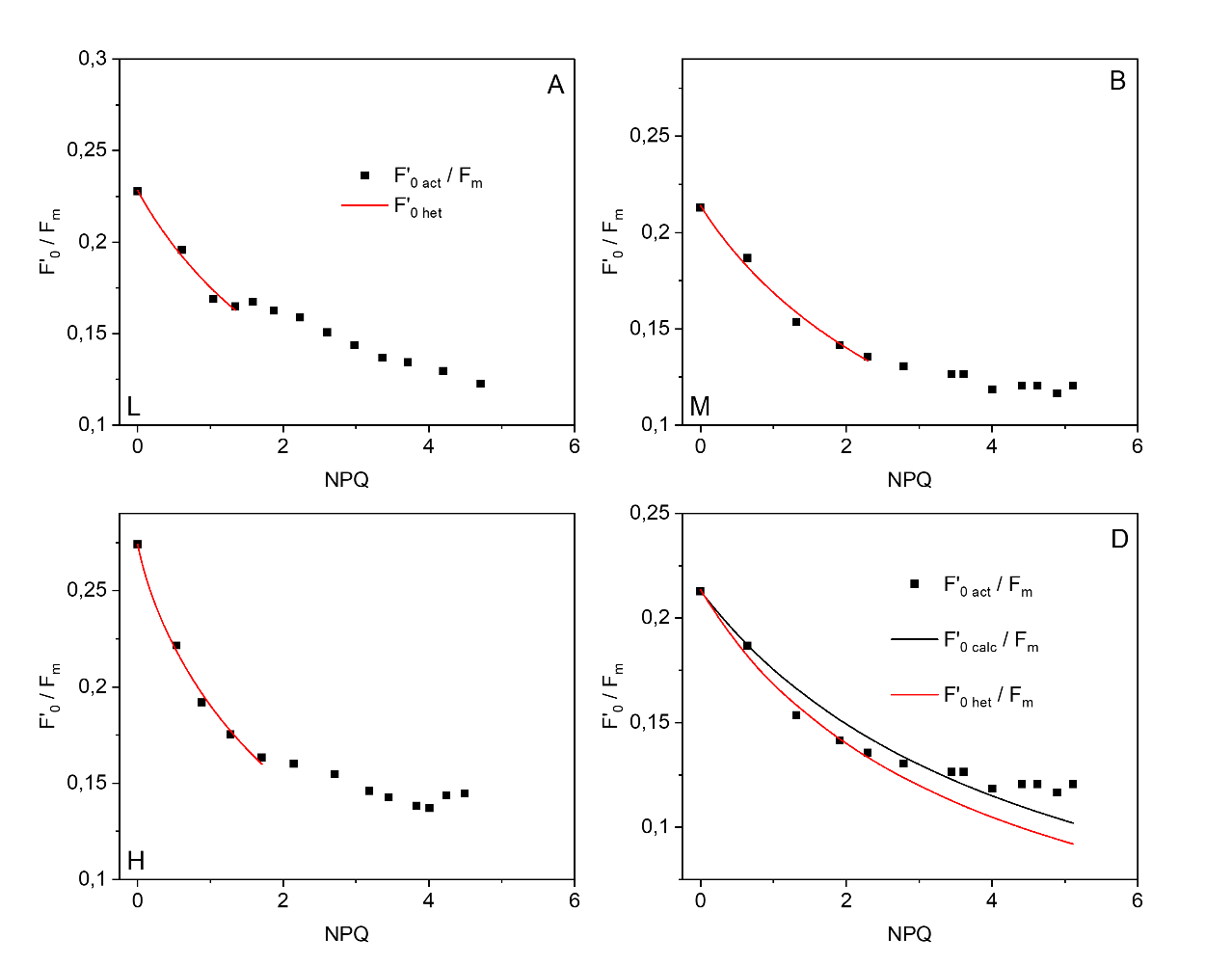


**Fig. S3**. Examples of minimum fluorescence as a function of Non-Photochemical Quenching (NPQ) in Selaginella martensii acclimated to deep shade (L), intermediate shade (M) and full sunlight (H). F’_0_ is expressed relative to its respective maximum fluorescence level (F_m_). (A-C) experimental, actual values of minimum fluorescence (F’_0_, black squares) together with the fitting function of F’_0_ accounting for a heterogeneous antenna (red line) in the three light regimes. The fitting procedure involves only the values of F’_0 act_ in the absence of photoinhibition (qP_d_>0.98), according to what postulated by Ware et al. (2015). Note that the subsequent F’_0 act_ drop as a function of NPQ is still approximately hyperbolic only in M and H plants (A-B), while the decrease in L plants is linear (C). (D) Example of F’_0 act_ (black squares), F’_0 calc_ (black line, homogeneous antenna, Oxborough and Baker 1997) and F’_0 het_ (red line, heterogeneous antenna, Ware et al. 2015) relative to the sample in (B).


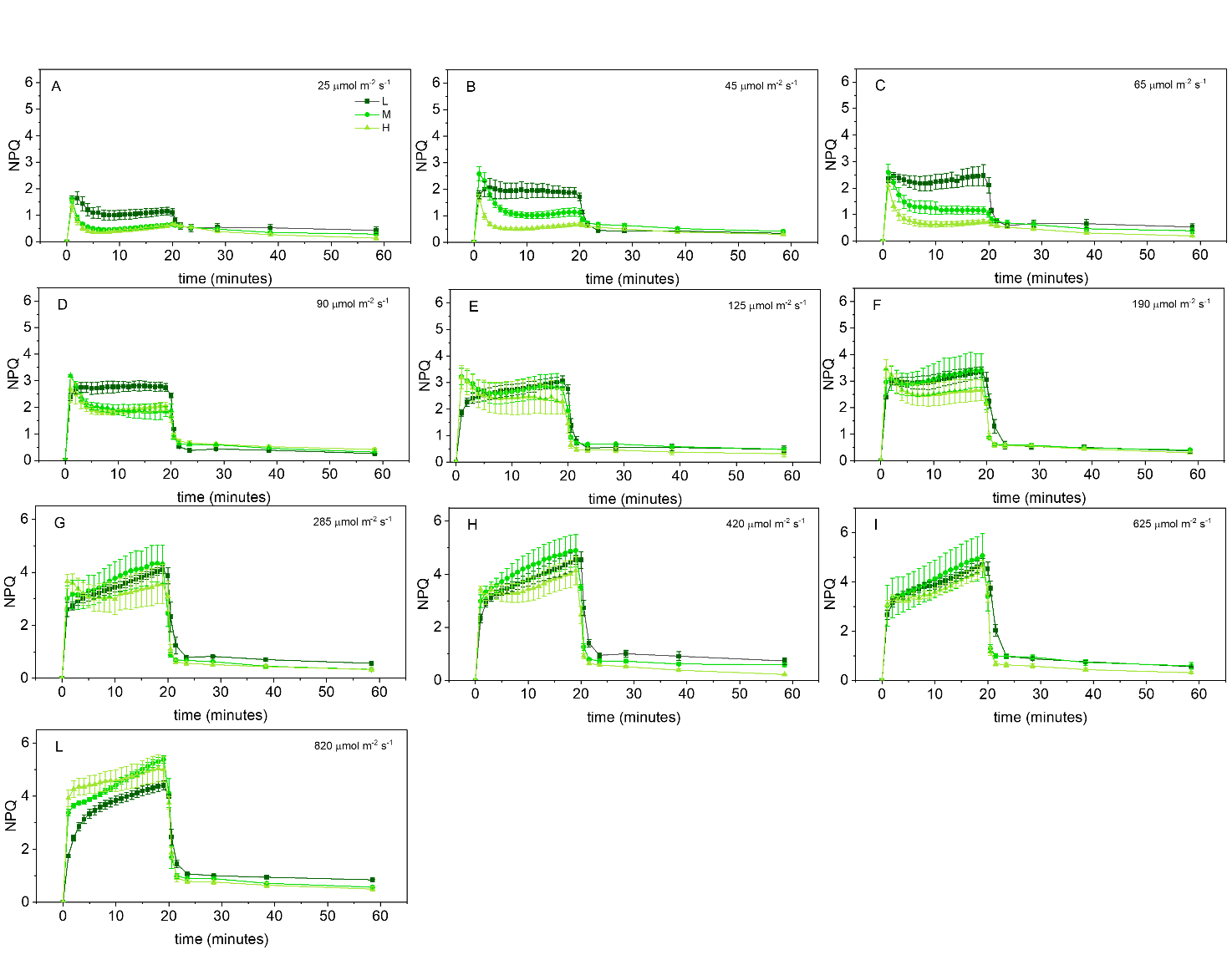


**Fig. S4**. Kinetic curves of NPQ during 19 minutes of continuous actinic light illumination followed by 40 minutes of dark relaxation in Selaginella martensii plants acclimated to deep shade (L), intermediate shade (M) and full sunlight (H). Levels of light intensity: 25, 45, 65, 90, 125, 190, 285, 420, 625, 820 µmol m^-2^ s^-1^. Average values ±Standard Error for n = 3-6.


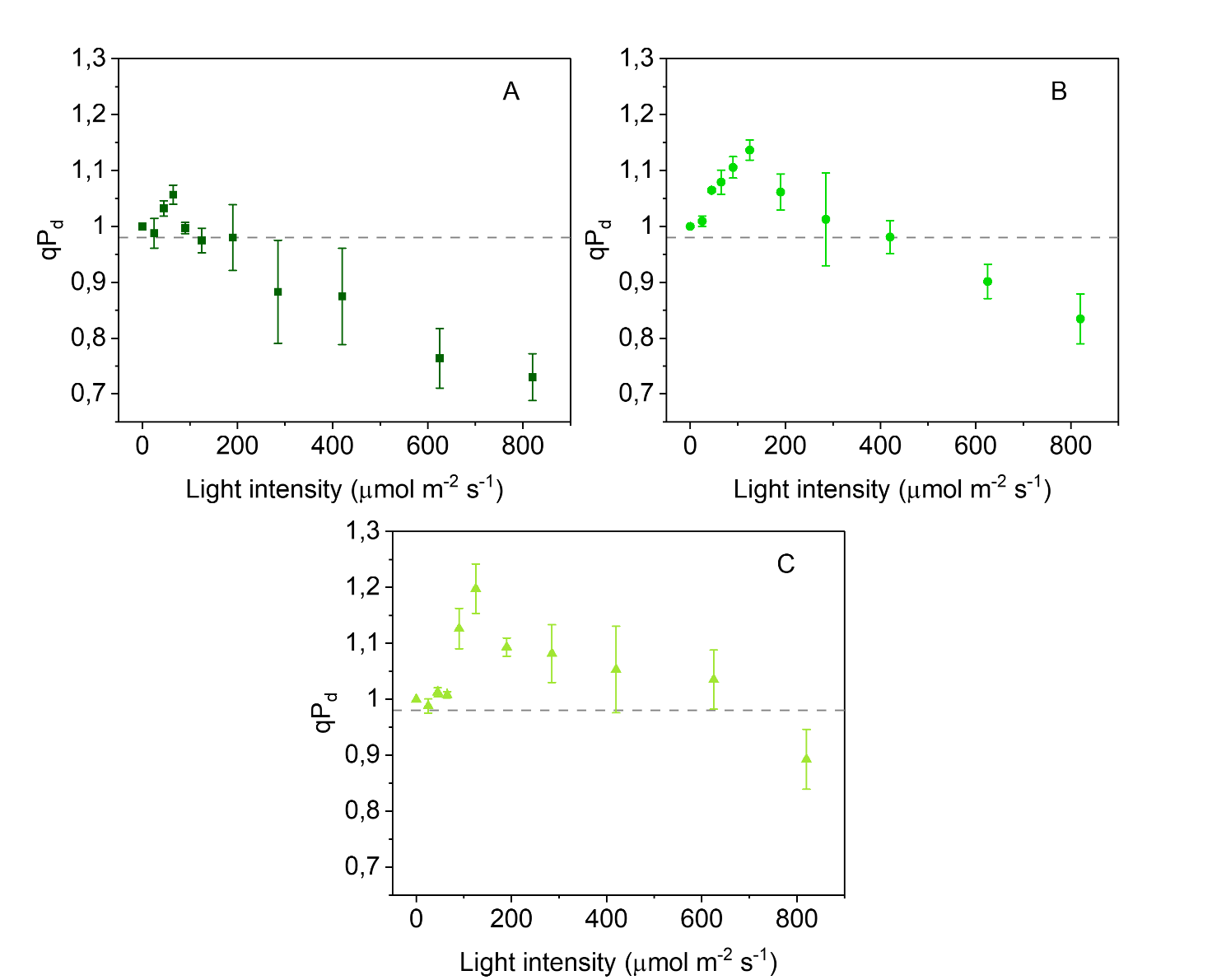


**Fig. S5.** Light curves of qP_d_ as function of light intensity. qP_d_ was calculated according to Ruban and Murchie (2012) in Selaginella martensii plants acclimated to deep shade (L), intermediate shade (M) and full sunlight (H). qP_d_ values were selected at specific times of the induction curve in order to closely match the number of photons that reached the samples at the end of each light interval of the light curve (the one treated in the paragraph “In sun plants higher photoprotection is accompanied by extensive PSII antenna uncoupling”)

References

Ferroni, L., Angeleri, M., Pantaleoni, L., Pagliano, C., Longoni, P., Marsano, F., et al. (2014). Light‐dependent reversible phosphorylation of the minor photosystem II antenna Lhcb6 (CP 24) occurs in lycophytes. *The Plant Journal*, 77(6), 893-905.

Järvi, S., Suorsa, M., Paakkarinen, V., & Aro, E.M. (2011). Optimized native gel systems for separation of thylakoid protein complexes: novel super-and mega-complexes. *Biochemical Journal*, 439(2), 207-214.

Oxborough, K., & Baker, N.R. (1997). Resolving chlorophyll a fluorescence images of photosynthetic efficiency into photochemical and non-photochemical components–calculation of qP and Fv/Fm; without measuring Fo’. *Photosynthesis Research*, 54(2), 135-142.

Ware, M.A., Belgio, E., & Ruban, A.V. (2015). Photoprotective capacity of non-photochemical quenching in plants acclimated to different light intensities. *Photosynthesis Research*, 126(2), 261-274.
